# Viral infection detection using metagenomics technology in six poultry farms of eastern China

**DOI:** 10.1371/journal.pone.0211553

**Published:** 2019-02-20

**Authors:** Yuan Qiu, Suchun Wang, Baoxu Huang, Huanxiang Zhong, Zihao Pan, Qingye Zhuang, Cheng Peng, Guangyu Hou, Kaicheng Wang

**Affiliations:** 1 China Animal Health and Epidemiology Center, Qingdao, Shandong, China; 2 Guangdong Institute of Applied Biological Resources, Guangzhou, Guangdong, China; 3 College of Veterinary Medicine, Nanjing Agricultural University, Nanjing, Jiangsu, China; WHO Collaborating Centre for Reference and Research on Influenza, AUSTRALIA

## Abstract

With rapidly increasing animal pathogen surveillance requirements, new technologies are needed for a comprehensive understanding of the roles of pathogens in the occurrence and development of animal diseases. We applied metagenomic technology to avian virus surveillance to study the main viruses infecting six poultry farms in two provinces in eastern China. Cloacal/throat double swabs were collected from 60 birds at each farm according to a random sampling method. The results showed that the method could simultaneously detect major viruses infecting farms, including avian influenza virus, infectious bronchitis virus, Newcastle disease virus, rotavirus G, duck hepatitis B virus, and avian leukemia virus subgroup J in several farms. The test results were consistent with the results from traditional polymerase chain reaction (PCR) or reverse transcription-PCR analyses. Five H9N2 and one H3N8 avian influenza viruses were detected at the farms and were identified as low pathogenic avian influenza viruses according to HA cleavage sites analysis. One detected Newcastle disease virus was classified as Class II genotype I and avirulent type according to F0 cleavage sites analysis. Three avian infectious bronchitis viruses were identified as 4/91, CK/CH/LSC/99I and TC07-2 genotypes by phylogenetic analysis of S1 genes. The viral infection surveillance method using metagenomics technology enables the monitoring of multiple viral infections, which allows the detection of main infectious viruses.

## Introduction

Animal disease surveillance is fundamental for animal disease prevention and control, and it is also a tool to get the information used to make decisions about control and eradication strategies. A variety of laboratory techniques have been used to survey the epidemiology of animal infectious diseases, among which animal pathogen surveillance and tracking the molecular epidemiology of pathogens are important strategies [1, 2]. These methods usually involve pathogen isolation and identification, reverse transcription-polymerase chain reaction (RT-PCR), real-time RT-PCR, and sequencing analyses. These methods often target a single known pathogen; however, the analysis of multiple pathogens in one or more hosts requires multiple detection methods, personnel, or even laboratories. Furthermore, these approaches cannot monitor and provide an early warning for pathogens of other animal diseases and viruses that have not yet been sequenced.

With the development of NGS technology, metagenomics, and bioinformatics, this problem has been solved gradually. Metagenomics has been used to detect and monitor human pathogens [3], animal pathogens [4], and plant pathogens [5]. Metagenomics-based detection methods are highly sensitivity, and they do not target any specific pathogens. Instead, they comprehensively scan the entire genome of the samples and, therefore, pathogens can be detected no matter whether they are included in the monitoring plan. Moreover, these methods can even discover the viruses which have not been sequenced, and they can directly analyze the genotypes, virulence, and molecular evolution of pathogens. The use of NGS has been confirmed for detecting microorganisms in human clinical samples [6–8] and infectious viruses in numerous animal species. The method of preparing the sequencing libraries and the NGS platform used in this study have been evaluated previously [9, 10], and viruses in clinical samples from single animal hosts have also been detected using the same standard NGS procedure in our laboratory [10–12].

In this study, we implemented viral infection surveillance in six poultry farms in two provinces of eastern China using short read sequencing with Ion Torrent and compared it with traditional viral infection surveillance methods.

## Materials and methods

### Ethics approval and consent to participate

This study was conducted according to the animal welfare guidelines of the World Organization for Animal Health and approved by the Animal Ethics Committee of China Animal Health and Epidemiology Center (CAHEC). The ethical board was provided by the Animal Ethics Committee of CAHEC (No.00501). Samples were collected with permission given by multiple relevant parties, including China Animal Health and Epidemiology Center, and the relevant farm owners.

### Sample collection

Six large-scale farms (more than 10,000 poultry per farm) comprising one chicken farm, one duck farm, and one goose farm in each of two provinces (Jiangsu and Anhui) in eastern China were selected at random. All the six large-scale poultry farms were enclosed farms for meat production. Cloacal/throat double swabs were collected from 60 live birds at each farm according to a random sampling method. The samples were stored in 0.5 mL phosphate buffered saline (PBS, pH 7.2) containing 10% glycerol at 4°C. Viral RNA extraction was performed within 3 days after collection. The remaining samples were stored at −80 °C.

### Viral RNA extraction

After sampling and mixing on a vortex mixer for 1 min, 20 μL of each of the 60 samples from each poultry farm were pooled. The pooled samples from the six farms were named AC, AD, AG, BC, BD, and BG. The meaning of the abbreviations AC, AD, AG, BC, BD and BG was showed in Table 1. The proportion of viral RNA was increased by pre-treating the samples, as described previously, with modifications [12]. The pooled samples were suspended on a vortex mixer for 1 min. and then clarified by centrifugation at 8,000g for 10 min. The supernatant was filtered through 0.22-μM filters to remove bacteria, and then treated with a mixture of 0.5 μL of recombinant DNase I (RNase-free, 5 U/μL) (Takara, Japan) and 0.5 μL ribonuclease A (RNase A, 10 mg/mL) (Takara, Japan) at 37 °C for 30 min to digest nucleic acids that were out of cells and virus particles. Subsequently, viral RNA was extracted using the QIAamp Viral RNA Mini Kit (Qiagen, Hilden, Germany) with loading each column six times and stored at −80 °C. Next Generation Sequencing (NGS) libraries were prepared and polymerase chain reaction (PCR)/reverse transcription-PCR (RT-PCR) were performed within 7 days.

**Table 1:** Sample details and sequencing quality.

Province Host	Jiangsu			Anhui		
	Chicken	Duck	Goose	Chicken	Duck	Goose
Sample name	AC	AD	AG	BC	BD	BG
Barcode	1	2	3	4	5	6
Number of reads	291,138	183,469	232,736	76,242	76,474	141,992
Average read length (bp)	145	139	134	117	142	136
≥Q20 bases	37,732,303	23,040,814	27,962,102	7,615,279	9,428,903	16,861,189
Percentage for the ≥Q20 bases (%)	89.38	90.35	89.66	85.37	86.83	87.31
Number of ≥Q20 reads	263,862	171,946	221,921	61,414	68,325	121,304
Percentage for the ≥Q20 reads (%)	90.63	93.72	95.35	80.55	89.34	85.43
GenBank ID	SRR5367358	SRR5367439	SRR5367441	SRR5367442	SRR5367444	SRR5367445
Number of reads matched to viruses including phages	1,886	227	16,503	6,295	35,993	58,315
The percentage of reads matched to viruses including phages (%)	0.71	0.13	7.44	10.25	52.68	48.07
Number of reads matched to viruses excluding phages	1,791	144	16,318	5,142	35,912	57,658
The percentage of reads matched to viruses excluding phages (%)	0.68	0.00	0.07	0.08	0.53	0.48
The percentage of reads matched to AIV in the total viral reads excluding phages (%)	96.31	15.28	100	99.55	99.91	100
The percentage of reads matched to IBV in the total viral reads excluding phages (%)	2.8	70.14	/	0.45	/	/
The percentage of reads matched to NDV in the total viral reads excluding phages (%)	/	/	/	/	0.09	/
The percentage of reads matched to Rotavirus in the total viral reads excluding phages (%)	0.67	/	/	/	/	/
The percentage of reads matched to DHBV in the total viral reads excluding phages (%)	/	14.58	/	/	/	/
The percentage of reads matched to ALV-J in the total viral reads excluding phages (%)	1.45	/	/	/	/	/
Number of bacteria reads	143,183	88,228	18,102	36,383	14,255	47,535
The percentage of bacteria reads (%)	54.26	51.31	8.16	59.24	20.86	39.19
Number of eukaryote reads	64,962	68,932	173,656	4,888	11,172	9,776
The percentage of eukaryote reads (%)	24.62	40.09	78.25	7.96	16.35	8.06
Number of unassigned reads	49,712	9,159	7,661	11,463	4,623	1,871
The percentage of unassigned reads (%)	18.84	5.33	3.45	18.66	6.77	1.54
Number of no-hit reads	4,119	5,400	5,999	2,385	2,282	3,807
The percentage of no-hit reads (%)	1.56	3.14	2.70	3.88	3.34	3.14

AIV: avian influenza virus; IBV: infectious bronchitis virus; NDV: Newcastle disease virus; DHBV: duck hepatitis B virus; ALV-J: J subgroup avian leukemia virus, HA: hemagglutinin; F: fusion protein: S: spike glycoprotein.

### NGS analysis of infectious viruses

Sequencing libraries of the extracted viral RNA from the pooled samples from the six farms (Table 1) were constructed using the Ion Total RNA-Seq kit v2 with some modifications [10]. Briefly, the extracted viral RNA was quantified by Qubit assays (Life Technologies, USA) to ensure samples were greater than 100ng/μL and then digested using RNase III. The fragmented RNA was ligated with random adaptors and barcodes, and this was followed by reverse transcription. The cDNA was amplified by PCR with 8 cycles, and amplicons of approximately 350 bp were collected with the E-Gel SizeSelect Agarose Gel (Life Technologies, USA). The six libraries were diluted to 26 pM and mixed with equal volumes. The Ion Personal Genome Machine (PGM) Template OT2 200 Kit (Life Technologies, USA) was used to pretreat the DNA libraries prior to sequencing. The treated samples were loaded onto an Ion 316 chip and sequenced using the Ion Torrent PGM platform (Life Technologies) with the Ion PGM Sequencing 200 Kit (Life Technologies, USA) [13]. Quality control was autorun by the Ion Torrent PGM platform to delete some low quality reads. The reads of the sequenced samples were compared to sequences in the non-redundant protein databases using the nucleotide Basic Local Alignment Search Tool (BLASTx) [14]. An E-value of 10^−5^ was used as the cutoff value for significant hits. The BLASTx results were parsed using the MetaGenome Analyzer (MEGAN version 5.10.5) with the default LCA parameters [15], and the taxonomic assignment was based on the first hit to identify the major viral infections in each farm.

### Verification of the infectious viruses

To verify the accuracy of the pathogen-detection results in the NGS analysis, the NGS results were compared with PCR or RT-PCR results. The AC, AD, AG, BC, BD, and BG samples were analyzed for the presence of the DNA or RNA of these viruses using the following detection methods. RT-PCR was performed using the PrimeScript One-step RT-PCR Kit Ver. 2 (Takara, Dalian, China; RR055A), and the reaction system included 12.5 μL of 2× One-step buffer, 1 μL each of the upstream and downstream primers (10 pM), 1 μL of PrimeScript 1 Step Enzyme Mix, 5 μL of nucleic acids, and 4.5 μL of RNase-free H_2_O. The reaction conditions were as follows: RT for 1 h at 50 °C, followed by 4 min at 95 °C, followed by 40 cycles of denaturation for 15 s, annealing for 30 s, and extension for 1 min at 72 °C, followed by a final extension for 7 min at 72 °C. PCR was performed using Premix *Taq* polymerase (version 2.0 plus dye) (RR901A, Takara); the reaction system included 12.5 μL of Premix *Taq*, 0.5 μL of upstream and downstream primers (10 pM), 0.5 μL of nucleic acids, and 25 μL of ultrapure water. The reaction conditions were as follows: 4 min at 95 °C, followed by 40 cycles of denaturation for 15 s, annealing for 30 s, and extension for 1 min at 72 °C, followed by a final extension for 7 min at 72 °C. Nucleic acids of the related viruses were also detected as positive controls. The amplified products were analyzed by agarose gel electrophoresis. The primers and their annealing temperatures in each test reaction are shown in Table 2.

**Table 2:** The PCR or RT-PCR methods used to verify the NGS results.

	Methods	Upstream primer (5’-3’)	Down- stream primer (5’-3’)	Annealing temperature (°C)	Target fragment length (bp)	Reference
**Detection**						
AIV	RT-PCR	TTCTAACCGAGGTCGAAAC	AAGCGTCTACGCTGCAGTCC	52	229	[16]
IBV	RT-PCR	TGAATCGTGGTAGGAGTG	AGCCCATCTGGTTGAAGT	50	409	[17]
NDV	RT-PCR	ATGGGCYCCAGAYCTTCTAC	CTGCCACTGCTAGTTGTGATAATCC	55	530	[18]
Rotavirus	RT-PCR	GTGCGGAAAGATGGAGAAC	GTTGGGGTACCAGGGATTAA	57	630	[19]
DHBV	PCR	ACTAGAAAACCTCGTGGACT	GGGAGAGGGGAGCCCGCACG	55	128	[20]
ALV-J	RT-PCR	GGATGAGGTGACTAAGA	CGAACCAAAGGTAACACACG	58	545	[21]
**Analysis**						
HA	RT-PCR	TATTCGTCTCAGGGAGCAAAAGCAGGGG	ATATCGTCTCGTATTAGTAGAAACAAGGGTGTTTT	45	1750	[22]
F	RT-PCR	TGTAGTAACGGGAGACAAAGCAG	GAATAAATACCAGAGACATAGGGA	46	957	[23]
S1	RT-PCR	ACTGAACAAAAGACMGACTTAGT	CCATAACTAACATAAGGGCAA	50	1700	[24]

PCR: polymerase chain reaction; RT-PCR: reverse transcription–polymerase chain reaction; AIV: avian influenza virus; IBV: infectious bronchitis virus; NDV: Newcastle disease virus; DHBV: duck hepatitis B virus; ALV-J: J subgroup avian leukemia virus, HA: hemagglutinin; F: fusion protein: S: spike glycoprotein.

### Avian influenza virus (AIV) subtype and pathotype analysis

The BLAST results of the NGS library sequences were opened in MEGAN, and the sequences of the avian influenza viruses detected in the six NGS libraries were extracted into respective FASTA files. Rapid Typing and Analysis Software of Influenza A Virus [25] conducted by China Animal Health and Epidemiology Center was used to analyze the extracted NGS sequences to determine the subtypes of the detected AIVs. Rapid Typing and Analysis Software of Influenza A Virus was established by Perl on Lunix systems to complete a series of analysis, typing and genetic evolution research of Influenza A Virus in short time, based on the sequence comparison results to the Influenza A Virus reference sequence including each gene in every subtype. The software has got the Computer Software Copyright Registration Certificate (No. 2804125) received from National Copyright Administration of the PRC. The phylogenetic relationship of the detected H9N2 AIVs were analyzed by amplifying the hemagglutinin (HA) genes (about 1700 bp) by RT-PCR using the reported specific primers [22] (Table 2). PCR products were purified using a QIAquick PCR purification kit (Qiagen, Inc.), cloned into the pMD18-T vector (Takara, Dalian, China), and then sequenced using synthetic oligonucleotides (Sangon Biotech, Shanghai, China). Nucleotide sequences of the RT-PCR products for HA genes obtained from the five H9N2 AIVs were aligned using MUSCLE software [26] and compared to the sequences of the other 28 reference H9 subtype isolates including all the H9 subtypes AIV lineages, using the software package MEGA 6.0 [27, 28]. The 28 reference isolates (S1 Table) included strains from four lineages (Clades h9.1-h9.4) of H9 subtype AIVs. The phylogenetic tree was calculated using the maximum likelihood (ML) method with the GTR+G substitution model and 1000 bootstraps. Their proteolytic cleavage sites (amino acids [1] 335–341 in H9N2 subtype and aa 342–348 in H3N8 subtype) [29, 30] in the translated HA amino acid sequence were analyzed to understand the pathogenicity of the AIVs [31].

### Infectious bronchitis virus (IBV) genotype and pathotype analysis

In recent researches, S1 gene sequencing and subsequent genetic analysis have provided a fast and accurate method for classifying and predicting genotypes of IBVs [32]. The S1 genes were amplified by RT-PCR using the reported primers [24] (Table 2). The PCR products were purified and cloned into the pMD18-T vector (Takara, Dalian, China), and then sequenced. The obtained nucleotide sequences of the RT-PCR products (about 1600 bp) and the deduced amino acid sequences of the IBV S1 genes were aligned using MUSCLE software [26]. The phylogenetic tree was calculated using the maximum likelihood (ML) method with the GTR+G+I substitution model and 1000 bootstraps, using the software package MEGA 6.0 [27, 28]. Thirty-three other IBV reference strains (S2 Table) for all the different genotypes were chosen for comparison with our isolates to identify the IBVs genotype [24].

### Newcastle disease virus (NDV) genotype and pathotype analysis

Hemagglutinin-neuraminidase (HN) gene segments were assembled from the NDV FASTQ sequence reads extracted by MEGAN, using CLC Genomics Workbench 8.5.1 (Qiagen). The reads used for assembly was mapped to the resulting contigs to identify if there are variants, indicative of multiple strains/genotypes present. The assembled HN fragment (about 450 bp) of the identified virus was then compared with those of 13 NDV strains (S3 Table) representative of all the different genotypes [33, 34] using MEGA 6.0 [28] to construct a phylogenetic tree and analyze the NDV genotypes. The phylogenetic tree was calculated using the maximum likelihood (ML) method with the GTR+I substitution model and 1000 bootstraps. Cleavage of the NDV F protein is known to be required for initiation of infection and is considered to be a major determinant of NDV virulence [35]. A fragment including the altered fusion protein (F) cleavage site was amplified using the reported primers F1 and F2 [23] (Table 2). PCR products were purified and cloned into the pMD18-T vector (Takara, Dalian, China), and then sequenced using synthetic oligonucleotides (Sangon Biotech, Shanghai, China). Their proteolytic cleavage sites (aa 112 to 118) in the translated amino acid sequence were analyzed to understand the pathogenicity of NDV[35, 36].

## Results

### Sequencing quality

The chip loading rate was 77%, and sequences with no barcode were removed. The quality threshold is 100 average 1-mer signal in library key. The minimum number of sequenced nucleotides was 8,906,866 bp and the maximum number was 42,132,207 bp. Sequencing data were deposited in GenBank under the accession numbers shown in Table 1. The proportion of reads that were classified as viral (compared to eukaryotic and bacterial), and of each virus identified in the total viral reads was listed in Table 1.

### Virus detection by NGS and verification

The NGS sequencing data were compared with the GenBank non-redundant protein databases, and the results showed that one to four species of viruses excluding phage were detected in each pool of samples (Table 3). AIV was detected in all the farms, and the results of the AIV subtype analysis using Rapid Typing and Analysis Software of Influenza A Virus are shown in Table 3. Five H9N2 and one H3N8 AIVs were detected in the farms. IBV was detected in three farms (chicken and duck farm in Jiangsu province, chicken farm in Anhui province). The chicken farm in Jiangsu province had the most kinds of viruses (four species, AIV, IBV, Rotavirus and avian leukemia virus), and a BLASTx analysis of reads located in env gene showed that the (ALV) in this farm belonged to subtype J, which was an endogenous ALV. The goose farm in Anhui province had the lowest number of detected viruses (one species, AIV subtype H9N2). NDV, Rotavirus G and duck hepatitis B virus were only detected in the duck farm in Anhui province, chicken and duck farms in Jiangsu province, respectively. The RT-PCR and PCR detection results of the six viruses in each farm are shown in Table 3, and the results (presence/absence) are consistent with the NGS results. All the viruses detected or undetected in NGS analysis were also present or absent in the RT-PCR and PCR verifications. The proportion of reads that were classified as viral (compared to eukaryotic and bacterial), and of each virus identified in the total viral reads was listed in Table 1.

**Table 3:** NGS and PCR (RT-PCR) detection of the viruses infecting animals at each farm.

Sample		NGS Detection Results /PCR (RT-PCR) Detection Results						AIV			NDV		IBV genotype
		AIV	IBV	NDV	Rotavirus	DHBV	ALV-J	Subtype	Lineage	HA cleavage site	Genotype	F0 cleavage site	
AC	Detection results	+/+	+/+	-/-	+/+	-/-	+/+	H9N2	H9.2.4.5	335 RSSR↓GLF 341	/	/	4/91
	No. of matched reads	1,725	50		12		26						
AD	Detection results	+/+	+/+	-/-	-/-	+/+	-/-	H3N8	/	342 KQTR↓GLF 348	/	/	TC07-2
	No. of matched reads	22	101			21							
AG	Detection results	+/+	-/-	-/-	-/-	-/-	-/-	H9N2	H9.2.4.5	335 RSSR↓GLF 341	/	/	/
	No. of matched reads	16,318											
BC	Detection results	+/+	+/+	-/-	-/-	-/-	-/-	H9N2	H9.2.4.5	335 RSSR↓GLF 341	/	/	CK/CH/LSC/99I
	No. of matched reads	5,119	23										
BD	Detection results	+/+	-/-	+/+	-/-	-/-	-/-	H9N2	H9.2.4.5	335 RSSR↓GLF 341	I	112 GKQGR↓LI118	/
	No. of matched reads	35,880		32									
BG	Detection results	+/+	-/-	-/-	-/-	-/-	-/-	H9N2	H9.2.4.5	335 RSSR↓GLF 341	/	/	/
	No. of matched reads	57,658											

NGS: Next Generation Sequencing; PCR: polymerase chain reaction; RT-PCR: reverse transcription–polymerase chain reaction; AIV: avian influenza virus; IBV: infectious bronchitis virus; NDV: Newcastle disease virus; DHBV: duck hepatitis B virus; ALV-J: J subgroup avian leukemia virus, HA: hemagglutinin.

### Viral infection analysis

**Fig 1:**
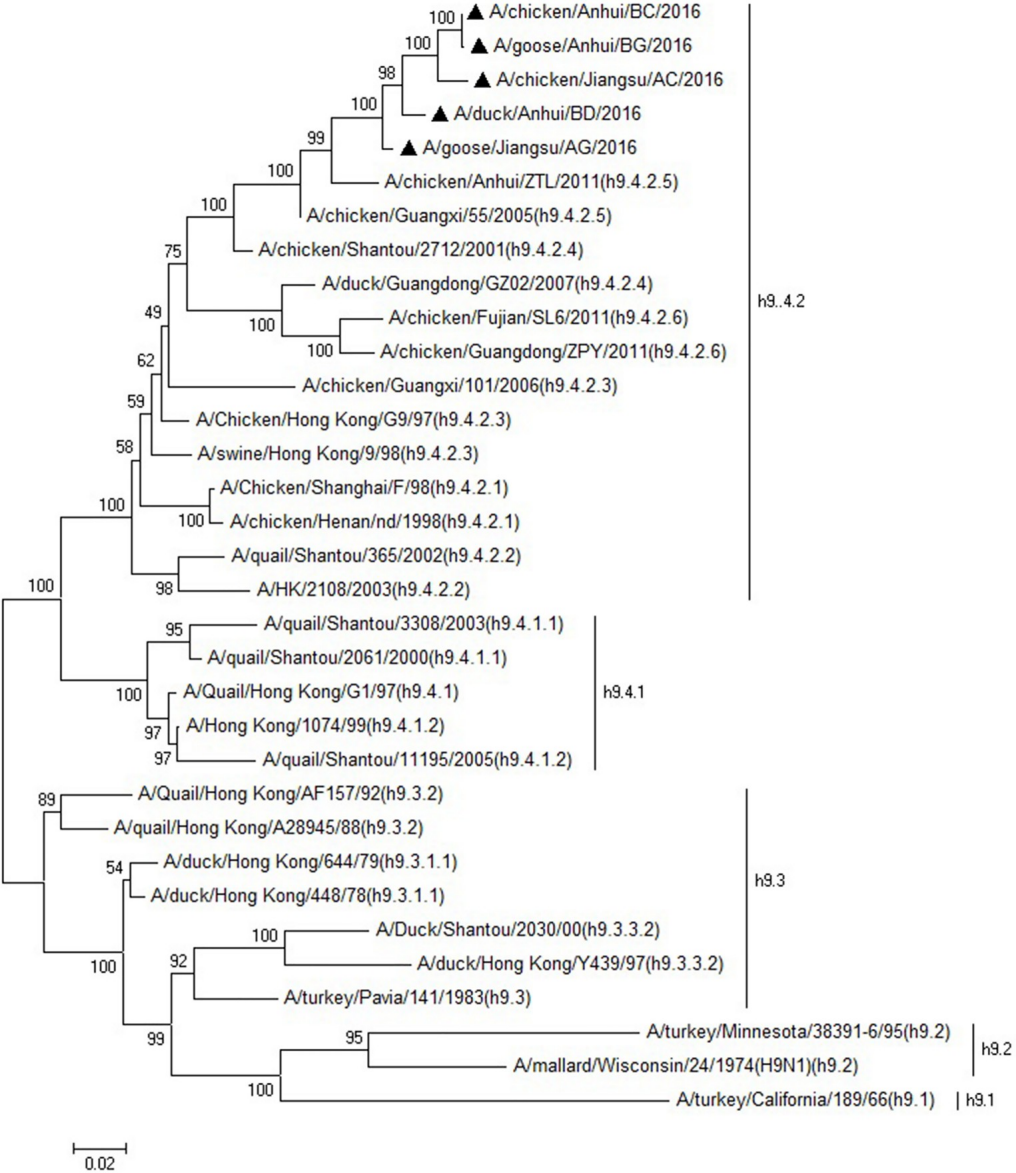
Phylogenetic analysis of the HA gene from 5 detected strains and 28 reference strains of H9 subtype AIVs. Nucleotide sequences of the RT-PCR products for HA genes (about 1700 bp) obtained from the five H9N2 AIVs were aligned using MUSCLE software and compared to the sequences of the other 28 reference H9 subtype isolates including all the H9 subtypes AIV lineages, using the software package MEGA 6.0. The 28 reference isolates included strains from four lineages (Clades h9.1-h9.4) of H9 subtype AIVs. The phylogenetic tree was calculated using the maximum likelihood (ML) method with the GTR+G substitution model and 1000 bootstraps. The scale bar indicates the branch length. ▲ AIVs detected by NGS in the poultry farms.

**Fig 2:**
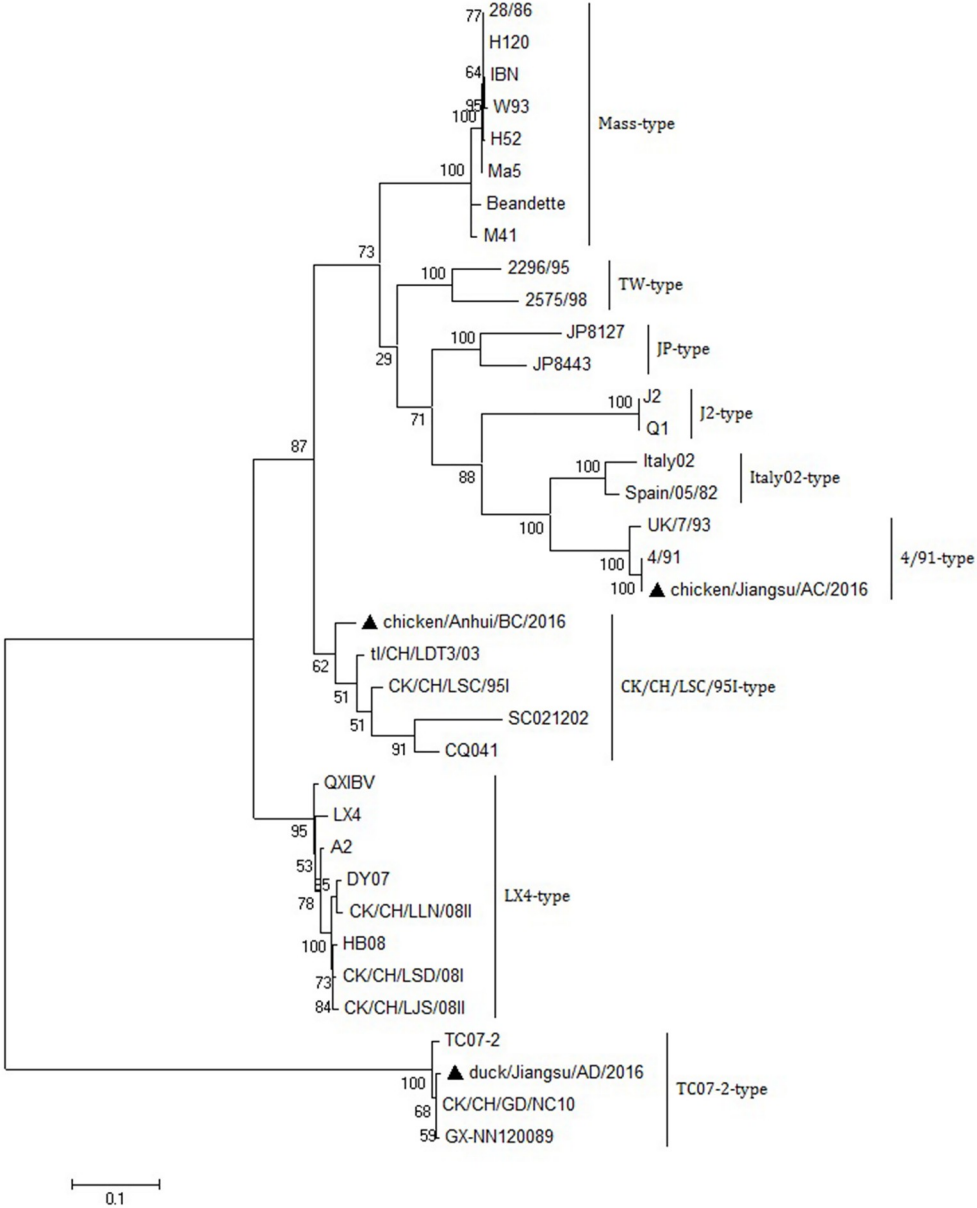
Phylogenetic analysis of the S1 genes from the 3 detected strains and 33 reference strains of IBVs. The sequences start at the AUG translation initiation codon. The obtained nucleotide sequences of the RT-PCR products (about 1600 bp) and the deduced amino acid sequences of the IBV S1 genes were aligned using MUSCLE software. The phylogenetic tree was calculated using the maximum likelihood (ML) method with the GTR+G+I substitution model and 1000 bootstraps, using the software package MEGA 6.0. The scale bar indicates the branch length. ▲ IBVs detected by NGS in the poultry farms.

**Fig 3:**
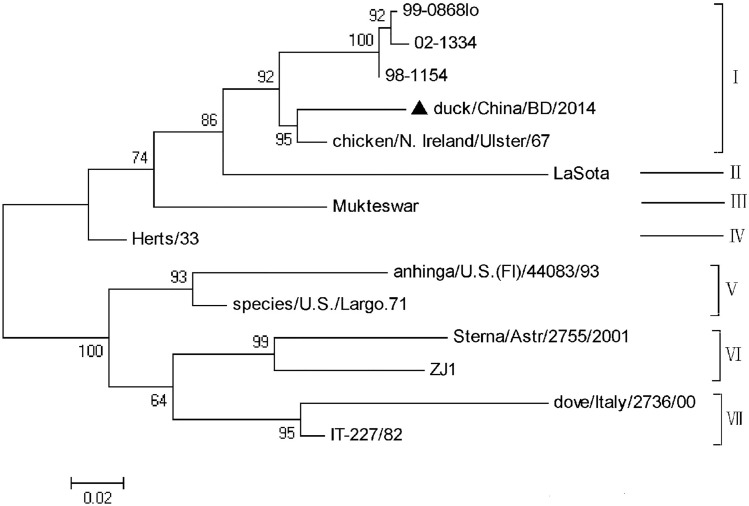
Analysis of the NDV genotypes detected in the duck farm in Anhui province. The assembled HN fragment (about 450 bp) of the identified virus was then compared with those of 13 NDV strains representative of all the different genotypes using MEGA 6.0 to construct a phylogenetic tree and analyze the NDV genotypes. The phylogenetic tree was calculated using the maximum likelihood (ML) method with the GTR+I substitution model and 1000 bootstraps. The scale bar indicates the branch length. ▲ NDV detected by NGS in the duck farm of Anhui province.

Phylogenetic analysis of the HA nucleotide sequences generated by PCR in the five H9N2 AIVs showed that all five detected H9N2 AIVs were in the h9.4.2.5 lineage (Fig 1), which is the current epidemic lineage in China [37]. Phylogenetic analysis of the S1 nucleotide sequences showed that all the 36 IBVs could be separated into nine genotypes (Fig 2), named LX4, CK/CH/LSC/99I, J2, 4/91, JP, TW, Italy02, TC07-2 and Mass. The three detected IBVs were identified as 4/91, CK/CH/LSC/99I and TC07-2 genotypes (Table 3). These three genotypes were also previously found in China [38], but had not been the most frequently isolated type (LX4) in China in recent years. The NDV detected in the duck farm in Anhui province was proven to be genotype I by HN gene analysis (Fig 3). Genotype I NDV has been detected in Chinese ducks in recent years [39]. Characterization of the proteolytic cleavage sites of the translated AIV HA and NDV F0 amino acid sequences are shown in Table 3. The results indicated that all the detected AIVs were low pathogenic viruses, and the NDV was avirulent.

The nucleotide sequences of the fragments amplified by RT-PCR in this study are available from GenBank under accession numbers MH487492–MH487500 and MH487502.

## Discussion

We used a metagenomics-based pathogen surveillance method to monitor viral infections in this study. The main infectious viruses in the six poultry farms were shown to be AIV and IBV, followed by NDV, ALV-J, DHBV, and rotavirus G, which is consistent with the current prevalence of poultry viral disease in China [40]. The high prevalence of viruses in the poultry farms could indicate insufficient farm biosecurity. We also analyzed the subtype of AIV, genotypes of NDV and IBV, and pathogenicities of AIV and NDV in this study. All the detected AIVs and NDV were low pathogenic or avirulent viruses. These kinds of viruses can be usually found in Chinese poultry farms [41, 42]. The three avian IBVs detected in the three farms were identified as genotypes 4/91, CK/CH/LSC/99I, and TC07-2, by phylogenetic analysis of S1 genes compared with 33 reference viruses. The TC07-2-type was first detected in Guangdong province in 2007 [43], and classified as CH VI lineage by S1 gene phylogenetic analysis [44]. In this study, the lineage was named as TC07-2-type, consistent with the principles of genotype nomenclature for molecular analysis of IBVs [45]. Because the assembled contigs from the NGS reads was too short (about 250 bp) or located in the genes which was not suitable for phylogenetic analysis, the phylogenetic analysis of AIV and IBV was based on the sequence of PCR fragments. Few reads matched to rotavirus G, DHBV, and ALV-J, and these matched reads were not located on genes available for phylogenetic analysis, and it was therefore not possible to construct phylogenetic trees for these three detected viruses. Because of the low abundance of viruses in the clinical samples, the quantity and quality of the viral sequence in the NGS should be enhanced to analyze the genotype and phylogenetic relationship from the NGS data directly. Because animals are fed in flocks, animal disease surveillance focuses on the infecting pathogens of each flock. Therefore, the metagenomics-based pathogen surveillance method established in this study also focused on poultry groups. The poultry in the same field were considered as a single sampling pool. The high sensitivity of the NGS technology ensures that major kinds of viral pathogens in mixed samples can be detected. In this study, the results were validated by traditional PCR or RT-PCR techniques, which were consistent with the metagenomics-based pathogen surveillance method to certify whether the viruses were percent or absent in the clinical samples. However, this method had some limitations. The absence of pathogens on farms where none were detected by NGS was not confirmed by comparing the current metagenomics results with an independent set of test results. Furthermore, no sequences other than the virus sequences were analyzed.

The detection results for the viruses were also related to the types of samples collected. We analyzed collected cloacal/throat double swabs, which could be used to detect major respiratory and gastrointestinal viruses, however, other types of infectious viruses, such as Marek’s disease virus, Egg drop syndrome virus, Infectious bursal disease virus and so on, could be detected in different types of samples, according to the monitoring purposes. H5 subtype AIV vaccine was used in all the six poultry farms. This study also analyzed the subtypes/genotypes and pathogenicities of the AIV, NDV and IBV strains, which were instructive for the development of better vaccines to control the respective diseases [46, 47].

Due to the high cost of NGS, only six samples were detected using the PGM platform in this study. However, despite the preliminary nature of this work used a small number of samples, it employed novel laboratory techniques for detecting animal infectious diseases detection. Once the cost of NGS falls, it will provide a routine detection method for testing large numbers of animal samples. These techniques will also aid the detection of viruses that have not yet been sequenced, which is critical for providing early warnings of disease outbreaks and the control and prevention of animal diseases.

In summary, we assessed a metagenomics-based pathogen surveillance method in this study. This method detected the main infectious viruses of poultry, and it analyzed the subtypes, genotypes and pathogenicity of some detected viruses. Thus, it is a useful method for animal disease surveillance. This method can also be used for animal disease detection, as well as to analyze disinfection procedures and other work in large-scale breeding fields, which can reduce the laboratory testing workload and enable a better understanding of infectious pathogens. This will provide important data for controlling animal infectious diseases.

## Supporting information

S1 Table The GenBank accession numbers of the published HA gene sequences in AIVs used in this study. (DOCX)

S2 Table The GenBank accession numbers of the published S1 gene sequences in IBVs used in this study. (DOCX)

S3 Table The GenBank accession numbers of the published HN gene sequences in NDVs used in this study. (DOCX)

## Abbreviations

AIV: avian influenza virus
ALV-J: J subgroup avian leukemia virus
BLAST: Basic Local Alignment Search Tool
DHBV: duck hepatitis B virus
F: fusion protein
HA: hemagglutinin
HN: hemagglutinin-neuraminidase
IBV: infectious bronchitis virus
MEGAN: MetaGenome Analyzer
NDV: Newcastle disease virus
NGS: next generation sequencing
PCR: polymerase chain reaction
RT-PCR: reverse transcription–polymerase chain reaction
S: spike glycoprotein
