# Supplementary material for: Viral infection detection using metagenomics technology in six poultry farms of eastern China
Source: PLoS One. 2019 Feb 20;14(2):e0211553. doi: 10.1371/journal.pone.0211553 (PMC6382132; doi:10.1371/journal.pone.0211553)
Supplement: S1 Table — (DOCX) [file pone.0211553.s001.docx]

**S1 Table. The GenBank accession numbers of the published HA gene sequences in AIVs used in this study.**

|  | Virus name | GenBank accession number |
| --- | --- | --- |
| 1 | A/turkey/California/189/66 | AF156390 |
| 2 | A/turkey/Minnesota/38391-6/95 | AF156387 |
| 3 | A/mallard/Wisconsin/24/1974(H9N1) | CY181337 |
| 4 | A/turkey/Pavia/141/1983 | KF188287 |
| 5 | A/duck/Hong_Kong/Y439/97 | AF156377 |
| 6 | A/Duck/Shantou/2030/00 | AF523390 |
| 7 | A/duck/Hong_Kong/644/79 | AY206678 |
| 8 | A/duck/Hong_Kong/448/78 | AB080224 |
| 9 | A/quail/Hong_Kong/A28945/88 | AY206675 |
| 10 | A/Quail/Hong_Kong/AF157/92 | AF156381 |
| 11 | A/Quail/Hong_Kong/G1/97 | AF156378 |
| 12 | A/quail/Shantou/3308/2003 | EF154949 |
| 13 | A/quail/Shantou/2061/2000 | EF154912 |
| 14 | A/Hong_Kong/1074/99 | AJ404627 |
| 15 | A/quail/Shantou/11195/2005 | EF154974 |
| 16 | A/Chicken/Shanghai/F/98 | AY743216 |
| 17 | A/chicken/Henan/nd/1998 | DQ997448 |
| 18 | A/quail/Shantou/365/2002 | EF154931 |
| 19 | A/HK/2108/2003 | DQ226106 |
| 20 | A/swine/Hong_Kong/9/98 | KX879589 |
| 21 | A/chicken/Guangxi/101/2006 | GU722363 |
| 22 | A/Chicken/Hong_Kong/G9/97 | AF156373 |
| 23 | A/duck/Guangdong/GZ02/2007 | EU926626 |
| 24 | A/chicken/Shantou/2712/2001 | CY024688 |
| 25 | A/chicken/Guangxi/55/2005 | EU086245 |
| 26 | A/chicken/Anhui/ZTL/2011 | JF715018 |
| 27 | A/chicken/Fujian/SL6/2011 | JF715052 |
| 28 | A/chicken/Guangdong/ZPY/2011 | JF715031 |
