# Supplementary material for: Viral infection detection using metagenomics technology in six poultry farms of eastern China
Source: PLoS One. 2019 Feb 20;14(2):e0211553. doi: 10.1371/journal.pone.0211553 (PMC6382132; doi:10.1371/journal.pone.0211553)
Supplement: S2 Table — (DOCX) [file pone.0211553.s002.docx]

**S2 Table. The GenBank accession numbers of the published S1 gene sequences in IBVs used in this study.**

|  | Virus name | GenBank accession number |
| --- | --- | --- |
| 1 | W93 | AY427818 |
| 2 | UK/7/93 | Z83979 |
| 3 | Spain/05/82 | DQ386104 |
| 4 | SC021202 | AY237817 |
| 5 | CQ041 | GQ265952 |
| 6 | QXIBV | AF193423 |
| 7 | LX4 | AY189157 |
| 8 | HB08 | GQ265934 |
| 9 | DY07 | GQ265927 |
| 10 | CK/CH/LSD/08I | GQ258336 |
| 11 | CK/CH/LLN/08II | GQ258323 |
| 12 | CK/CH/LJS/08II | GQ258321 |
| 13 | A2 | AY043312 |
| 14 | Q1 | AF286302 |
| 15 | Ma5 | KU736747 |
| 16 | M41 | DQ830980 |
| 17 | JP8443 | AY296745 |
| 18 | JP8127 | AY296744 |
| 19 | J2 | AF286303 |
| 20 | Italy02 | AJ457137 |
| 21 | IBN | AY856348 |
| 22 | H52 | AF352315 |
| 23 | H120 | KU736750 |
| 24 | Beandette | AJ311317 |
| 25 | 4/91 | AF093793 |
| 26 | 28/86 | AY846750 |
| 27 | 2575/98 | AY606314 |
| 28 | 2296/95 | AY606321 |
| 29 | CK/CH/LSC/95I | DQ167146 |
| 30 | tl/CH/LDT3/03 | KT852992 |
| 31 | CK/CH/GD/NC10 | HQ018903 |
| 32 | GX-NN120089 | KJ999805 |
| 33 | TC07-2 | GQ265948 |
