# Supplementary material for: Viral infection detection using metagenomics technology in six poultry farms of eastern China
Source: PLoS One. 2019 Feb 20;14(2):e0211553. doi: 10.1371/journal.pone.0211553 (PMC6382132; doi:10.1371/journal.pone.0211553)
Supplement: S3 Table — (DOCX) [file pone.0211553.s003.docx]

**S3 Table. The GenBank accession numbers of the published HN gene sequences in NDVs used in this study.**

|  | Virus name | GenBank accession number |
| --- | --- | --- |
| 1 | Sterna/Astr/2755/2001 | AY865652 |
| 2 | ZJ1 | AF431744 |
| 3 | 99-0868lo | AY935496 |
| 4 | 02-1334 | AY935490 |
| 5 | 98-1154 | AY935491 |
| 6 | dove/Italy/2736/00 | GQ429293 |
| 7 | chicken/N._Ireland/Ulster/67 | AY562991 |
| 8 | IT-227/82 | AJ880277 |
| 9 | Mukteswar | EF201805 |
| 10 | anhinga/U.S.(Fl)/44083/93 | AY562986 |
| 11 | species/U.S./Largo.71 | AY562990 |
| 12 | LaSota | AF077761 |
| 13 | Herts/33 | AY741404 |
